# The microbiome in inflammatory bowel diseases: from pathogenesis to therapy

**DOI:** 10.1007/s13238-020-00745-3

**Published:** 2020-06-29

**Authors:** Sheng Liu, Wenjing Zhao, Ping Lan, Xiangyu Mou

**Affiliations:** Guangdong Provincial Key Laboratory of Colorectal and Pelvic Floor Diseases, The Sixth Affiliated Hospital, School of Medicine, Sun Yat-sen University, Guangzhou, 510275 China

**Keywords:** inflammatory bowel disease, pathogenesis, etiology, microbiome, dysbiosis, therapy

## Abstract

Inflammatory bowel disease (IBD) has become a global disease with accelerating incidence worldwide in the 21st century while its accurate etiology remains unclear. In the past decade, gut microbiota dysbiosis has consistently been associated with IBD. Although many IBD-associated dysbiosis have not been proven to be a cause or an effect of IBD, it is often hypothesized that at least some of alteration in microbiome is protective or causative. In this article, we selectively reviewed the hypothesis supported by both association studies in human and pathogenesis studies in biological models. Specifically, we reviewed the potential protective bacterial pathways and species against IBD, as well as the potential causative bacterial pathways and species of IBD. We also reviewed the potential roles of some members of mycobiome and virome in IBD. Lastly, we covered the current status of therapeutic approaches targeting microbiome, which is a promising strategy to alleviate and cure this inflammatory disease.

## INTRODUCTION

Inflammatory bowel disease (IBD), including ulcerative colitis (UC) and Crohn’s disease (CD), has become a global disease with accelerating incidence worldwide in the 21st century (Ng et al., 2018). IBD is characterized by chronic immune-mediated intestinal inflammation that is driven by genetic susceptibility, environmental and microbial factors (Ni et al., 2017; Imhann et al., 2018).

Microbial factors have been historically proven to be indispensable for the onset of IBD (Alhagamhmad et al., 2016) and advances in high-throughput sequencing has enabled us to elucidate the gut microbiome in IBD. Study of microbial etiology of IBD has been mainly focused on three directions: 1) the persistent pathogen theory 2) the excessive bacterial translocation theory and 3) the dysbiosis theory (De Hertogh et al., 2008; Kalischuk and Buret, 2010). The persistent pathogen theory hypothesizes IBD can be caused by persistent infection of an enteric pathogen like *Mycobacterium avium* subspecies *paratuberculosis*, *Clostridium difficile*, and adhesion-invasive *Escherichia coli* (AIEC). The excessive bacterial translocation theory suggests the excessive level of translocation of intestinal bacteria across the intestinal barrier is a cause of IBD. While, the dysbiosis theory hypothesizes that the shift of balance between “beneficial” vs. “detrimental” commensal bacteria can cause IBD.

The three theories are not mutually exclusive. For example, AIEC can be considered as both a persistent pathogen and detrimental commensal bacteria. The first two theories were comprehensively reviewed elsewhere (De Hertogh et al., 2008; Kalischuk and Buret, 2010); while in this review, we summarize the emerging evidences that imply the roles of dysbiosis in pathogenesis of IBD and the potential therapeutic options that target the gut microbiome to alleviate IBD.

## POTENTIAL ROLES OF DYSBIOSIS IN PATHOGENESIS OF IBD

IBD is characterized by chronic immune-mediated intestinal inflammation that attacks the bowel. IBD has been consistently shown to be associated with gut dysbiosis (Kostic et al., 2014; Lynch and Pedersen, 2016). Although many IBD-associated dysbiosis have not been proven to be a cause or an effect of IBD, it is often hypothesized that at least some of alteration in microbiome is protective or causative.

Metagenomic studies have revealed microbial compositional changes in patients with IBD (Franzosa et al., 2019; Lloyd-Price et al., 2019) and metabolomic studies have revealed many defined microbial metabolites are depleted in individuals with IBD versus control individuals (Franzosa et al., 2019). Some of the depleted metabolites and related species are found to have anti-inflammatory effects and therefore are hypothesized to be protective; on the other hand, pro-inflammatory bacterial metabolites and species that are enriched in IBD patients are hypothesized to be causative, in terms of IBD.

In this review, we mainly focus on hypothesis that has both types of supporting evidences: 1) at least one association study in human IBD (rather than animal models); and 2) at least one pathogensis study in human, animal models, or cell models that explains the result in the association study.

### Potentially protective bacterial pathways and species

The metabolic pathways encoded by the human gut microbiome produce numerous bioactive molecules that interact with the host. Typical bioactive molecules include short-chain fatty acids (SCFAs) and tryptophan derivatives that are produced by bacteria from dietary components, as well as secondary bile acids (BAs) that are bacteria-modified host products (Postler and Ghosh, 2017).

SCFAs (primarily acetate, propionate, and butyrate) produced by gut bacteria regulates protective immunity and reduces tissue inflammation (Furusawa et al., 2013; Kim et al., 2013). One study found that 12% of metabolic pathways were significantly different between IBD patients and healthy controls, and it confirmed a decrease in butanoate and propanoate metabolism genes in CD (Morgan et al., 2012). Another case-control analysis using shotgun metagenomic sequencing of stool samples from 1,792 individuals suggests the fermentation of pyruvate to butanoate, a butyrate precursor, was decreased in patients with IBD (Vich Vila et al., 2018). When looking at the bacterial species composition, a decreased amount of the commensal bacterium *Faecalibacterium prausnitzii* was reported in IBD patients compared with controls (Sokol et al., 2009; Hedin et al., 2016; Lloyd-Price et al., 2019). *In vitro* peripheral blood mononuclear cell stimulation by *F*. *prausnitzii* led to significantly lower IL-12 and IFN-γ production levels and higher secretion of the anti-inflammatory cytokine IL-10 (Sokol et al., 2008b). Additionally, various *F*. *prausnitzii* isolates have abilities to simulate IL-10 secretion by dendritic cells (DCs) (Rossi et al., 2016), which suggests the anti-inflammatory role of *F*. *prausnitzii* in colitis. A 15 kDa protein with anti-inflammatory properties, produced by *F*. *prausnitzii*, could alleviate colitis in mice by inhibiting the NF-κB pathway (Quevrain et al., 2016). Some *F*. *prausnitzii* strains are considered as candidates of next-generation probiotics (Martin et al., 2017).

*Roseburia* were also significantly reduced in IBD (Morgan et al., 2012), and IBD-genetic risk score was significantly associated with a decrease of *Roseburia* in healthy controls (Imhann et al., 2018). The depletion *Roseburia hominis* was observed in CD (Franzosa et al., 2019; Lloyd-Price et al., 2019) and UC (Machiels et al., 2014). In stool samples from patients with CD or UC, the strain abundance of *Roseburia intestinalis* decreased (Vich Vila et al., 2018). *R*. *intestinalis* are further reported as acetate-to-butyrate converters that reside in the intestinal mucus layer, where their anti-inflammatory effects may occur (Vich Vila et al., 2018).

In addition to reduced SCFA levels, decreased tryptophan metabolism levels were associated with a compromised epithelial barrier in IBD (Schirmer et al., 2019). Tryptophan can be converted by bacteria into bioactive indole-containing molecules that activate the aryl hydrocarbon receptor and down-regulates inflammation (Zelante et al., 2013). Indoleacrylic acid that promotes mucus production and suppresses inflammatory cytokine production was found reduced in patients with IBD (Wlodarska et al., 2017). Tryptophan-metabolizing pathways have been identified in some members of the human gut microbiota such as *Clostridium sporogenes* and *E*. *coli* (Williams et al., 2014; Dodd et al., 2017; Agus et al., 2018).

Primary BAs (PBAs) are produced by the host and then modified by bacteria into secondary BAs (SBAs) which mainly have anti-inflammatory activities. A normal bacterial BA metabolism plays an important role in modulating the host regulatory T (Treg) cell homeostasis (Song et al., 2020), as well as TH17 and Treg cell differentiation (Hang et al., 2019). Disrupted BAs metabolism has been observed in IBD patients, with fecal BAs pools skewed toward decreased SBAs and increased PBAs relative to healthy controls (Duboc et al., 2013; Franzosa et al., 2019). A more recent study also found that fecal BAs composition was altered (dominated by PBAs) in a sub-group of CD patients who did not sustain remission (Connors et al., 2019). PBAs cholate and its glycine and taurine conjugates were enriched in dysbiotic samples from participants with CD, and by contrast, the SBAs lithocholate and deoxycholate were reduced in dysbiosis (Lloyd-Price et al., 2019). Moreover, levels of lithocholic acid and deoxycholic acid (the most abundant gut SBAs), and genes required to convert PBAs to SBAs were reduced in stool from UC relative to familial adenomatous polyposis (FAP) (Sinha et al., 2020). Members of the *Roseburia* and unclassified *Subdoligranulum* species were associated with BAs metabolism, and they were both markedly reduced in IBD (Lloyd-Price et al., 2019). Ruminococcaceae was reduced in IBD compared to healthy people (Vich Vila et al., 2018; Lo Presti et al., 2019; Yilmaz et al., 2019) and is known to contain members (particularly *Clostridium leptum*) capable of SBAs generation, which ameliorate intestinal inflammation in a process reliant on the TGR5 bile acid receptor (Sinha et al., 2020). Supplementation of SBAs also reduces intestinal inflammation in three murine colitis models (Sinha et al., 2020).

Polysaccharide A (PSA) produced by *Bacteroides fragilis* directs the development of CD4^+^ T cells and induces the anti-inflammatory function of Tregs (Mazmanian et al., 2005; Round et al., 2011). PSA protects animals from experimental colitis depending on IL-10-producing CD41 T cells (Mazmanian et al., 2008). Individuals with IBD had a significantly lower percentage of the *B*. *fragilis* population with PSA promoter orientated “ON” (Blandford et al., 2019). Moreover, sphingolipids produced by *B*. *fragilis* regulate homeostasis of host intestinal natural killer T cells and confer protection against oxazolone-induced experimental colitis (An et al., 2014).

Recent research on *Akkermansia muciniphila* revealed another potential protective pathway against IBD. Initially, a study of 46 IBD and 20 control patients showed that the abundance of *Akkermansia muciniphila* reduced many fold in CD and in UC (Png et al., 2010). Although a contradictory research indicated *A*. *muciniphila* was sufficient for promoting intestinal inflammation in both specific-pathogen-free and germ-free IL10(− / −) mice model of IBD (Seregin et al., 2017), in a follow-up study, *A*. *muciniphila* strain ATCC BAA-835 was examined in gnotobiotic IL10(−/−) mice, and it did not promote short-term intestinal inflammation (Ring et al., 2019). *A*. *muciniphila* was shown to improve the gut barrier partially via its outer membrane protein Amuc_1100 that interacts with Toll-like receptor 2 (Plovier et al., 2017)*.* The roles of *A*. *muciniphila* in modulating human immunological homeostatic was further demonstrated by the recent report that *A*. *muciniphila* induce homeostatic IgG production and antigen-specific T cell responses in mice (Ansaldo et al., 2019) and that *A*. *muciniphila* treatment ameliorated Dextran Sulfate Sodium (DSS)-induced UC in mice (Bian et al., 2019).

### Potential causative bacterial pathways and species

The integrated human microbiome project has revealed a few metabolites, notably nicotinuric acid, taurine, and acylcarnitines are more abundant in IBD patients than controls (Lloyd-Price et al., 2019). Interestingly, taurine has been previously identified as a mucosal inflammasome activator (Levy et al., 2015). Therefore, these metabolites were suggested as potential causative metabolites and therapeutic targets. The acylcarnitines-related species are *Roseburia hominis*, *Klebsiella pneumoniae, Haemophilus parainfluenzae*, and *Clostridium bolteae* (Lloyd-Price et al., 2019)*.* Bacterial genes with virulence-related functions were enriched in IBD patients (Erickson et al., 2012; Morgan et al., 2012), presumably due to overgrowth of functionally altered commensals termed pathobionts. *Escherichia coli* revealed an increased amount in patients with IBD (Lloyd-Price et al., 2019; Pittayanon et al., 2019) and the adherent invasive *E*. *coli* (AIEC) pathovar are associated specifically with ileal mucosa in IBD (Darfeuille-Michaud et al., 2004; Sepehri et al., 2011), suggesting AIEC may contribute to IBD pathogenesis (Mylonaki et al., 2005; Garrett et al., 2010).

The frequent recovery of *E*. *coli* adhering to intestinal mucosa of patients with CD (Darfeuille-Michaud, 2002; Martin et al., 2004; Prorok-Hamon et al., 2014) and UC (Kotlowski et al., 2007) has stimulated great interest. Interaction of AIEC with intestinal mucosa in the context of IBD include: (1) AIEC cross the mucous layer and resist antimicrobial peptides; (2) AIEC adhere to intestinal epithelial cells (IECs) via FimH and carcinoembryonic antigen related cell adhesion molecule 6 (CEACAM6), and lead to colonisation of the gut mucosa; (3) AIEC enter lamina propria and Peyer’s patches across M cells via long polar fimbriae (LPF) expression, and interact with immune cells (Palmela et al., 2018). AIEC can promote inflammation, survive and replicate, and escape autophagy when inside macrophages (Bringer et al., 2006). Besides, AIEC also have the ability to evade the host immune response by suppressing IFN-γ mediated signal transducer and activator of STAT1 in IECs, preventing an appropriate antimicrobial response (Ossa et al., 2013). AIEC strain NC101 harbors the *pks* pathogenicity island that encodes the biosynthetic machinery for synthesizing the genotoxin colibactin (Nougayrede et al., 2006). Monocolonization with the commensal NC101 promoted invasive carcinoma and tumorigenesis in azoxymethane-treated IL-10(−/−) mice (Arthur et al., 2012; Eaton et al., 2018).

In a recent study, an *Enterococcus faecium* strain that has adhesion gene was isolated from the feces of UC patients, promotes colitis and colonic cytokine expression (Seishima et al., 2019). A previous research showed colonic inflammation in IL10(−/−) mice inoculated with *Enterococcus faecalis* and *faecium* strains is associated with gene expression changes similar to those of human IBD (Barnett et al., 2010).

Another pathobiont that associated with IBD is enterotoxigenic *Bacteroides fragilis* (ETBF) (Prindiville et al., 2000; Zamani et al., 2017). ETBF induces focal colonic Stat3 activation and Th17 immune responses and then promotes mucosal permeability (Wick et al., 2014; Chung et al., 2018; Dejea et al., 2018). Genes for *B*. *fragilis* toxin (*bft*) encode secreted oncotoxins, and increase IL-17 in the colon (Dejea et al., 2018). Besides promoting IBD, ETBF are also possibly driving FAP and CRC (Thiele Orberg et al., 2017; Garrett, 2019).

*Campylobacter concisus* is another adherent, invasive proteobacterium that has been associated with IBD (Zhang et al., 2009; Man et al., 2010; Mahendran et al., 2011; Mukhopadhya et al., 2011; Kirk et al., 2016; Underwood et al., 2016). Although the natural colonization site of *C*. *concisus* is oral cavity, *C*. *concisus* can also colonize the intestinal tract. Intestinal colonization by bacteria from the oral cavity has been suggested to be extensively involved in inflammatory diseases (Cao, 2017; Dickson, 2018). Some *C*. *concisus* strains acquired zonula occludens toxin (*zot*) gene from a phage, and increased intestinal membrane permeability by affecting the tight junctions (Zhang et al., 2014). *C*. *concisus* Zot may have enteric pathogenic potential by damaging intestinal epithelial barrier, inducing intestinal epithelial and macrophage production of proinflammatory cytokines in particular TNF-α (Mahendran et al., 2016), thus triggering the relapse of IBD. *C*. *concisus* cause epithelial sodium channel dysfunction via IL-32-regulated ERK1/2, as well as claudin-8-dependent barrier dysfunction, both of which contribute to Na(+) malabsorption and enteritis (Nattramilarasu et al., 2020).

Another oral cavity and gastrointestinal bacterium, *Fusobacterium varium,* may be one of the pathogenic factors in UC. *F*. *varium* bacteria were present at a higher abundance in the colonic mucosa of patients with UC compared to healthy controls (Ohkusa et al., 2002). When administered by rectal enema in mice, *F*. *varium* was able to cause colonic mucosal inflammation (Ohkusa et al., 2003). *F*. *varium* invaded host intestinal epithelial cells, significantly increased the concentrations of IL-8 and TNF-α, and triggered host inflammatory reactions (Ohkusa et al., 2009). Genome analysis of a *F*. *varium* strain showed it possesses multiple virulence factors, including type V secretion system (T5SS) and *Fusobacterium* adhesion (FadA) paralogs, which involve in potential mucosal inflammation (Sekizuka et al., 2017).

*Ruminococcus gnavus* is part of the healthy gut microbiota in humans, but it is enriched in IBD (Png et al., 2010; Willing et al., 2010; Joossens et al., 2011; Nishino et al., 2018; Franzosa et al., 2019; Lloyd-Price et al., 2019). The increased level of *R*. *gnavus* has also been linked to spondyloarthritis (Breban et al., 2017), pouchitis in UC patients who have undergone a total colectomy (Machiels et al., 2017), and allergic diseases (Chua et al., 2018). 199 strain-specific genes involved in oxidative stress responses, adhesion, iron-acquisition, and mucus utilization were identified, potentially conferring an adaptive advantage for the *R*. *gnavus* clade in the IBD gut (Hall et al., 2017). *R*. *gnavus* produce and metabolize 2,7-anhydro-Neu5Ac to achieve nutritional competitive advantage in mucus against other bacteria (Tailford et al., 2015; Owen et al., 2017; Bell et al., 2019). In addition, *R*. *gnavus* synthesizes and secretes a pro-inflammatory complex polysaccharide, which potently induces TNF-α secretion by DCs via TLR4 (Henke et al., 2019).

Non*-pylori Helicobacter* also has numerous associations with IBD. In a cross-sectional study of 73 CD and 92 controls, CD is associated with the presence of enterohepatic *Helicobacter* spp. species DNA in intestinal biopsies (Laharie et al., 2009). Enterohepatic *Helicobacter* including *H*. *hepaticus* (Kullberg et al., 2001, 2006; Yang et al., 2013) and *H*. *bilis* (Jergens et al., 2007; Liu et al., 2011; Atherly et al., 2016) are often referred as pathobionts (Chai et al., 2017), because they have been shown to be capable of causing IBD-like disease in mice. *H*. *hepaticus* predominantly induces inflammatory TH17 cells in disease-susceptible IL-10-deficient animals and contributes to spontaneous colitis (Xu et al., 2018). *Helicobacter pylori* infection was reported to be negative associated with IBD (el-Omar et al., 1994; Sonnenberg and Genta, 2012; Rokkas et al., 2015), supporting a possible protective benefit of *H*. *pylori* infection against the development of IBD. Alternatively, IBD could be a protective factor against *H*. *pylori* infection. The presence of IBD-associated gastric mucosal lesions may create an inhospitable environment for *H*. *pylori* colonization (Castano-Rodriguez et al., 2017).

### Dysbiosis in mycobiome

Besides bacterial dysbiosis, alterations in the eukaryotic fungal community (the “mycobiome”) are also important. Fungal composition in IBD is characterized with an increased Basidiomycota/Ascomycota ratio (Sokol et al., 2017), which was also skewed with higher values in CRC than control (Coker et al., 2019).

*Candida* spp. is significantly more abundant in patients with CD (Li et al., 2014; Lam et al., 2019) or IBD (Chehoud et al., 2015). In particular, *Candida albicans* were enriched in CD (Li et al., 2014), UC (Mar et al., 2016), as well as the general IBD patients (Sokol et al., 2017). *Candida tropicalis* are pathogenic fungus found in mouse intestine and when SPF mice were colonized with them, *Clec7a*(−/−) mice developed much severe colitis compared with uncolonized *Clec7a*(−/−) mice or colonized WT mice (Iliev et al., 2012; Tang et al., 2015). These findings suggest that fungal dysbiosis is associated with IBD and that *Candida* species are consistently associated with the inflamed gut (Li et al., 2019). In addition, a common skin resident fungus *Malassezia restricta* is specifically abundant in CD patients, and exacerbates colitis in mouse models through mechanisms requiring CARD9, a signaling protein involved in antifungal immunity (Limon et al., 2019).

Additionally, there was a decreased proportion of *Saccharomyces cerevisiae* compared with healthy subjects in IBD (Sokol et al., 2017), and *S*. *cerevisiae* were depleted in CRC (Coker et al., 2019). *S*. *cerevisiae* UFMG A-905 showed protective potential in a murine model of acute UC (Tiago et al., 2015). *S*. *cerevisiae* CNCM I-3856 had been shown to reduce AIEC-induced ileal colitis in a mouse model, by inhibiting AIEC adhesion to enterocytes and restoring barrier function (Sivignon et al., 2015). In another study, however, *S*. *cerevisiae* colonization exacerbated intestinal disease and increased gut barrier permeability in a mouse model of colitis (Chiaro et al., 2017).

### Dysbiosis in virome

Enteric virome is mainly consisted of bacteriophages. Among IBD subjects, the changes in virome composition reflected alterations in bacterial composition (Clooney et al., 2019). *Caudovirales* phage sequences were detected in intestinal washes and biopsy tissues of Australian pediatric CD patients (Wagner et al., 2013), and they were also observed in IBD patients from a UK cohort and two US validation cohorts (Norman et al., 2015). The abundance of intestinal *Caudovirales* phage families, including *Siphoviridae*, *Myoviridae* and *Podoviridae*, were elevated in a mouse model of colitis (Duerkop et al., 2018). Recent study showed phages from active UC patients induced more IFN-γ via a TLR9-dependent pathway, which is linked to aggravated intestinal inflammation and colitis (Gogokhia et al., 2019), suggesting that certain phages may trigger intestinal inflammation in the gut and contribute to IBD.

## THERAPEUTIC APPROACHES TARGETING MICROBIOME

### Probiotics, prebiotics and postbiotics

Probiotics are defined as live microorganisms which when administered in adequate amounts confer a health benefit on the host (Hill et al., 2014). Prebiotic is a substrate that is selectively utilized by probiotics conferring a health benefit (Gibson et al., 2017), while postbiotic is referring a bioactive molecule produced by a probiotic. American Gastroenterological Association Institute advised that probiotics may be considered for treatment of functional symptoms in IBD (Colombel et al., 2019). Probiotics could induce anti-inflammatory effects, improve (or restore) barrier function, and beneficially modulate the composition of the microbiome by inhibiting the growth of detrimental bacteria and promoting the growth of beneficial species (Abraham and Quigley, 2017).

As a prebiotic, inulin acts on IBD by retaining microbial populations, supporting epithelial barrier function, and defending against invasion and pathogens translocation (Akram et al., 2019).The probiotic cocktail VSL#3 (a mix of 4 lactobacilli, 3 bifidobacteria and 1 strain of *Streptococcus*) reduced recurrence and maintain remission in patients with CD (Fedorak et al., 2015) and UC (Bibiloni et al., 2005; Miele et al., 2009). A meta-analysis showed that probiotic cocktail VSL#3 was effective in inducing remission of active UC, and the probiotics may be as effective as 5-aminosalicylates (5-ASAs) in preventing relapse of quiescent UC (Derwa et al., 2017). Moreover, probiotic *Lactobacillus reuteri* ATCC 55730 (Oliva et al., 2012), and *E*. *coli* strain Nissle 1917 (Scaldaferri et al., 2016; Sonnenborn, 2016) also have shown efficacy in the treatment of UC; however, a percentage of adverse events such as diarrhea and abdominal pain were reported in patients treated with *E*. *coli* strain Nissle 1917 (Kruis et al., 2004; Sassone-Corsi et al., 2016). In contrast, *Lactobacillus acidophilus* La-5 and *Bifidobacterium animalis subsp*. *lactis* BB-12 (Probio-Tec AB-25) was demonstrated with no significant clinical benefit in comparison with placebo for maintaining remission in patients with UC (Wildt et al., 2011). Additionally, several randomized, double-blind trials indicated administration of *Lactobacillus rhamnosus* in children with gastroenteritis did not have better outcomes than those who received placebo (Freedman et al., 2018; LaMont, 2018; Schnadower et al., 2018). More recently, a multi-strain probiotic (*Lactobacillus rhamnosus* NCIMB 30174, *Lactobacillus plantarum* NCIMB 30173, *Lactobacillus acidophilus* NCIMB 30175 and *Enterococcus faecium* NCIMB 30176) is associated with decreased intestinal inflammation in patients with UC, but not with CD (Bjarnason et al., 2019).

In addition to the traditional probiotics mentioned above, next-generation probiotics (NGPs) including *F*. *prausnitzii* and *A*. *muciniphila* were proposed (O’Toole et al., 2017). Oral administration of either live *F*. *prausnitzii* or its supernatant (containing its postbiotics) markedly reduced the severity of TNBS colitis, partly due to secreted metabolites able to block NF-κB activation and IL-8 production (Sokol et al., 2008a). C57BL/6 male mice administered *A*. *muciniphila* once daily by oral gavage for 14 days improved DSS-induced colitis, which was evidenced by colon length shortening, histopathology scores and enhanced barrier function (Bian et al., 2019). *A*. *muciniphila* or a specific outer membrane protein Amuc_1100 (as a postbiotic) blunted colitis, with a reduction in infiltrating macrophages and CD8(+) cytotoxic T lymphocytes (CTLs) in the colon (Wang et al., 2020). In addition to looking for new probiotic species, synthetic biology techniques were used to improve existing probiotics. A recent research showed an *E*. *coli* Nissle 1917 strain, engineered to secrete the curli-fused trefoil factors, promotes intestinal barrier function and epithelial restitution, and enhance protective effects against colitis in mice (Praveschotinunt et al., 2019).

### Phage therapy

Phages are highly specific and typically lyse a subgroup of strains within one bacterial species, indicating they have a limited impact on the overall composition of the subject’s microbiome and are likely to have a better safety profile than antibiotic therapy. A randomized trial of oral T4-like coliphages or a commercial Russian coliphage product therapy in 120 children with acute bacterial diarrhea in Bangladesh did not report any adverse events but failed to improve diarrhea outcome (Sarker et al., 2016). Another two clinical trials also showed oral bacteriophage are safe in healthy children and adults (McCallin et al., 2013; Sarker et al., 2017). Though they demonstrated the safety feature of phage therapy, more knowledge is needed on *in vivo* phage-bacterium interaction and assessing the efficacy in reducing severity of gastrointestinal diseases.

In recent years, phage therapy has re-gained attention as a therapeutic approach to combat infectious disease and non-communicable diseases. For instance, engineered phages was used for a human mycobacterial infection that are resistant to antibiotics (Dedrick et al., 2019), and phages that specifically targets cytolytic *E*. *faecalis* attenuated alcoholic liver disease in a recent study (Duan et al., 2019). AIEC are abnormally predominant on the ileal mucosa of IBD patients, and they bind to the CEACAM6 receptor expressed on the surface of epithelial cells (Barnich et al., 2007). Bacteriophages that targets AIEC reduced DSS-induced colitis symptoms on AIEC strain LF82-colonised CEABAC10 transgenic mice, expressing the human CEACAM6 receptor for AIEC, and significantly decreased the number of AIEC in faeces and in the adherent flora of intestinal sections (Galtier et al., 2017). Therefore, phages targeting AIEC strains are a promising new treatment for IBD.

### Fecal microbiota transplantation (FMT)

FMT, where fecal microbiota from a healthy donor is transplanted into a patient’s GI tract, is already a successful therapy for recurrent *Clostridium difficile* infection (CDI) (Hamilton et al., 2012; van Nood et al., 2013; Hvas et al., 2019). The prevailing hypothesis is that FMT might correct the dysbiosis associated with IBD, leading to a restoration of the gut microbial homeostasis (Burrello et al., 2018). The restored colon microbial community could inhibit *C*. *difficile* by multiple mechanisms: competition for nutrients; direct suppression by antimicrobial peptides; bile-acid-mediated inhibition of spore germination and vegetative growth; and activation of immune-mediated colonization resistance (Khoruts and Sadowsky, 2016).

It also has received extended attention in the treatment of CD (Zhang et al., 2013; Cui et al., 2015) and UC (Moayyedi et al., 2015; Paramsothy et al., 2017a, 2019). Improved remission rates for patients treated with FMT, possibly dependent on donor fecal composition, the use of multiple FMTs, and early treatment (Moayyedi et al., 2015). FMT appears effective in UC remission induction, but long-term durability and safety remain unclear (Paramsothy et al., 2017b). A significant fraction of patients with recurrent CDI have IBD, and FMT is somewhat less effective in clearing CDI from patients with IBD compared with patients without IBD (Khoruts et al., 2016).

Some key issues should be followed: FMT indications; donor selection; preparation of faecal material; clinical management and faecal delivery; registries, monitoring of outcomes and ethical issues; basic requirements for implementing an FMT centre (Cammarota et al., 2017, 2019). Moreover, the gut fungal together with viral community in donor stool may affect the FMT outcome of treating IBD. It has been reported high abundance of *Candida albicans* in donor stool reduce FMT efficacy in CDI (Zuo et al., 2018). And when studying viral transfer following FMT, multiple recipients from a single donor displayed highly individualised virus colonisation patterns (Draper et al., 2018).

## CONCLUSION AND PERSPECTIVE

**Figure 1:**
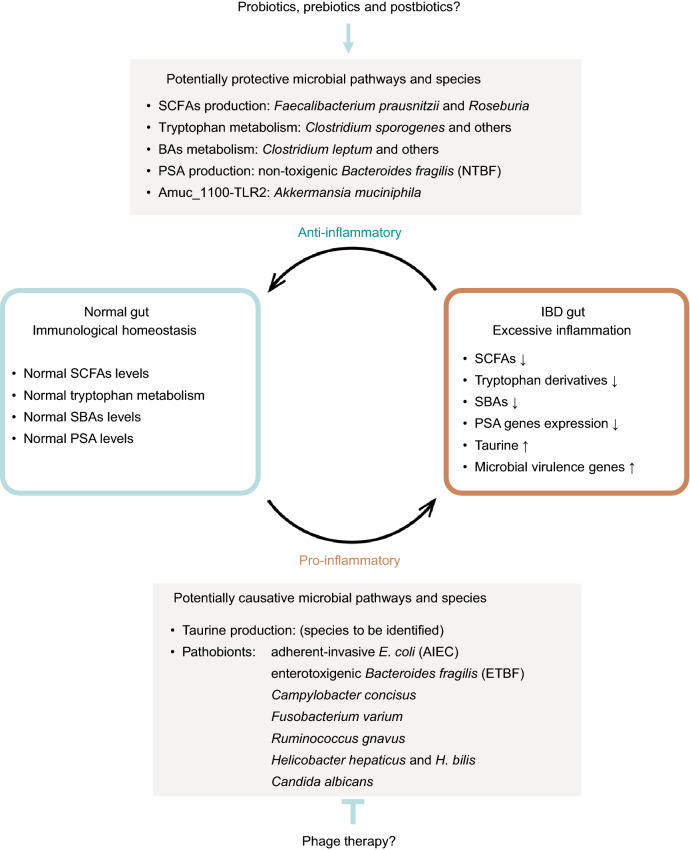
**Graphical summary of potentially protective and causative microbial bacterial pathways and species in IBD.** SCFAs, tryptophan derivatives, secondary BAs and PSA gene expression are found depleted in human IBD gut. They are also proved to have anti-inflammatory effects in biological models and therefore are often proposed as protective factors. Prebiotics, probiotics or postbiotics targeting these factors are promising strategies to alleviate IBD. In contrast, taurine is found enriched in the metabolome and virulent genes are found enriched in microbiome of human IBD gut. Taurine and the virulence-gene-containing pathobionts also have pro-inflammatory effects in biological models and therefore are proposed as potentially causative factor for IBD. Phage therapies that target these factors are promising strategies to alleviate IBD. SCFAs, short-chain fatty acids; SBAs, secondary bile acids; PSA, polysaccharide A

In the past decade there have been major advances in pathogenesis, pharmacological, and surgical interventions for both UC and CD. Current clinical applications for IBD diagnosis and treatment has been extensively reviewed by British Society of Gastroenterology consensus, and it highlights the importance of multidisciplinary research (Lamb et al., 2019). In this review we summarized the potential protective and causative microbial pathways and species in IBD (Fig. 1) as well as current status of therapeutic approaches targeting microbiome. Understanding of dysbiosis and the microbial pathways of specific microorganisms has suggested multiple strategies for modifying the intestinal microbiota to prevent or ameliorate IBD.

Some symbiotic organisms such as AIEC, *Campylobacter concisus*, *Fusobacterium varium*, *Ruminococcus gnavus*, and *Helicobacter* species, are often referred to as pathobionts because they may cause disease under certain conditions. For AIEC, potential therapeutic strategies include targeting bacterial colonization of gut mucosa, such as the use of phage therapy, bacteriocins and anti-adhesion molecules. *Bacteroides fragilis* produce PSA and sphingolipids, which regulate homeostasis and protect animals from experimental colitis. In contrast, ETBF induce IL-17 in the colon and DNA damage in colonic epithelium, promoting IBD or even FAP and CRC.

Precise targeting of the metabolic pathways that are used by harmful bacteria may provide a new strategy to treat IBD. For example, tungstate-mediated microbiota editing reduced the severity of intestinal inflammation in mouse models of colitis (Zhu et al., 2018), and oral administration of sodium tungstate inhibited molybdoenzymes selectively decreased gut colonization with genotoxin-producing *Enterobacteriaceae*, thus reducing carcinogenesis in mouse models of colitis-associated CRC (Zhu et al., 2019).

Prebiotics are promising approaches to modify human microbiome. In the context of IBD, dietary fibers promote a selected group of SCFA-producing strains and regulate BAs profiles. SCFAs, particularly butyrate, promote the development of Treg cells and mucus production to down-regulate inflammatory signaling pathways and to strengthen the epithelial barrier. Restoration of SCFA producers by selected dietary fibers is a promising approach for managing IBD.

Traditional probiotics including *Bacillus* spp., *Bifidobacterium* spp., *Lactobacillus* spp., and *S*. *cerevisiae* have showed variably ameliorative effects on IBD; however, the number of patients in these trails are relatively small. Additionally, the major challenge of utilization traditional probiotics is that we do not understand the precise probiotic mechanisms of these bacteria in the context of IBD. In contrast, next-generation probiotics (NGPs or sometimes called live biotherapeutics) are based on the outcomes of mechanism studies. Some *F*. *prausnitzii*, *Roseburia* and *A*. *muciniphila* strains represent promising next-generation probiotic candidates. Notably, a normal bacterial BAs metabolism, especially SBAs, also play an important role modulating the host immunological homeostasis. The therapeutic potential of *Clostridium sporogens* and other SBA producing-species in IBD warrants additional investigations.

The safety of phages targeting intestinal pathogens is well documented for adults and children, based on data for several clinical trials in which no adverse events were reported. This is not surprising as phages are the most abundant viruses present in the human gastrointestinal tract. Phage-mediated targeting of *E*. *faecalis* ameliorated alcoholic liver disease, indicating precisely editing the intestinal microbiome is another promising direction. It would be interesting to test whether IBD could be treated by phages that target potentially causative bacteria reviewed in this article including AIEC. Besides bacteria, *Candida* species are consistently more abundant in IBD. Mycophage that targets *Candida* species may inhibit their colonization and contribute to the alleviation of IBD.

FMT can be used as a therapeutic option to treat CDI in the context of IBD when first line antibiotics are ineffective. The impact of phage on microbial dynamics is a factor that should be considered. *Caudovirales* phage are more significantly enriched in the intestine of individuals with IBD, which supports the notion that elevated *Caudovirales* phages might predict FMT failure and need for additional maintenance FMT delivery or escalation of treatment. Furthermore, *Candida albicans*, the fungal community that are more abundant in IBD patients compared to healthy individuals, compromises FMT efficacy in a mouse model of CDI. Therefore, further research is needed to explore whether pre-FMT eradication of *C*. *albicans* in some recipients might increase FMT success rates in some cases. US FDA recently issued a safety alert about the potential risk of transmission of pathogenic bacteria by FMT products and the resultant serious adverse reactions that may occur. It’s important to implement Shiga toxin-producing *E*. *coli* and enteropathogenic *E*. *coli* screening into the quality and safety protocols. Overall, FMT shows some evidence of benefit in IBD; however, it should only be used in the context of clinical trials until further high-quality evidence clarifies optimal administration protocol.

To conclude, we are excited to see the recent advances in microbiome research in IBD and anticipate studies in IBD pathogenesis provide more insights to facilitate therapeutic efforts to ameliorate this increasingly common disease.
